# Lipopolysaccharide-Induced Neuronal Activation in the Paraventricular and Dorsomedial Hypothalamus Depends on Ambient Temperature

**DOI:** 10.1371/journal.pone.0075733

**Published:** 2013-09-19

**Authors:** Samuel P. Wanner, Kyoko Yoshida, Vladimir A. Kulchitsky, Andrei I. Ivanov, Kazuyuki Kanosue, Andrej A. Romanovsky

**Affiliations:** 1 Systemic Inflammation Laboratory (FeverLab), Trauma Research, St. Joseph's Hospital and Medical Center, Phoenix, Arizona, United States of America; 2 School of Physical Education, Physiotherapy and Occupational Therapy, Federal University of Minas Gerais, Belo Horizonte, Brazil; 3 Department of Physiology, School of Allied Health Sciences, Faculty of Medicine, Osaka University, Osaka, Japan; 4 Institute of Physiology, National Academy of Sciences, Minsk, Belarus; 5 Department of Human and Molecular Genetics and Virginia Institute of Molecular Medicine, Virginia Commonwealth University, Richmond, Virginia, United States of America; 6 Graduate School of Sport Sciences, Waseda University, Tokorozawa, Japan; 7 Interdisciplinary Graduate Program in Neuroscience, Arizona State University, Tempe, Arizona, United States of America; Rosalind Franklin University, United States of America

## Abstract

Systemic inflammatory response syndrome is associated with either fever or hypothermia, but the mechanisms responsible for switching from one to the other are unknown. In experimental animals, systemic inflammation is often induced by bacterial lipopolysaccharide (LPS). To identify the diencephalic and brainstem structures involved in the fever-hypothermia switch, we studied the expression of c-Fos protein, a marker of neuronal activation, in rats treated with the same high dose of LPS (0.5 mg/kg, intravenously) either in a thermoneutral (30°C) or cool (24°C) environment. At 30°C, LPS caused fever; at 24°C, the same dose caused profound hypothermia. Both fever and hypothermia were associated with the induction of c-Fos in many brain areas, including several structures of the anterior preoptic, paraventricular, lateral, and dorsal hypothalamus, the bed nucleus of the *stria terminalis*, the posterior pretectal nucleus, ventrolateral periaqueductal gray, lateral parabrachial nucleus, *area postrema*, and nucleus of the solitary tract. Every brain area studied showed a comparable response to LPS at the two different ambient temperatures used, with the exception of two areas: the dorsomedial hypothalamic nucleus (DMH), which we studied together with the adjacent dorsal hypothalamic area (DA), and the paraventricular hypothalamic nucleus (PVH). Both structures had much stronger c-Fos expression during LPS hypothermia than during fever. We propose that PVH and DMH/DA neurons are involved in a circuit, which – depending on the ambient temperature – determines whether the thermoregulatory response to bacterial LPS will be fever or hypothermia.

## Introduction

Systemic inflammation is strongly associated with changes in the regulation of deep body temperature (T_b_). Whereas a majority of patients with systemic inflammatory response syndrome have an increased T_b_, or fever, about 10% of patients (those with most severe inflammation) present a lowered T_b_, or hypothermia [1], [2]. The same thermoregulatory manifestations, fever and hypothermia, occur in animal models of systemic inflammation. In the laboratory, aseptic systemic inflammation is often induced by injecting rats intravenously or intraperitoneally with lipopolysaccharide (LPS, a cell-wall constituent of gram-negative bacteria). When the dose of LPS is low (<100 µg/kg), and when the thermal environment is neutral or warm, rats respond to LPS with a fever; when the dose is high, and when the thermal environment is subneutral, the predominant response is hypothermia [2]–[5]. Although some autonomic and behavioral thermoeffectors [6], [7] and biochemical processes [8] have been reported to be selectively involved in LPS hypothermia (as opposed to LPS fever), what determines the fever-hypothermia switch remains unknown, and the neuroanatomical substrate of this switch has not been elucidated. In an attempt to identify the diencephalic and brainstem structures involved in the switch between fever and hypothermia, we studied the brain distribution of c-Fos protein, a marker of rapid cellular activation, in rats challenged with the same high dose of LPS in either a thermoneutral environment (in which the typical response is fever) or cool environment (in which the predominant response is hypothermia). We found two hypothalamic structures in which the Fos response to LPS depended strongly on the thermal environment.

## Methods

### Animals and surgery

The experiments were conducted in male Wistar rats (Harlan, Indianapolis, IN, USA) in accordance with the recommendations in the Guide for the Care and Use of Laboratory Animals of the National Institutes of Health and under protocols approved by the St. Joseph's Hospital Animal Care and Use Committee. The rats were housed individually in cages in a rack equipped with a Smart Bio-Pack ventilation system and Thermo-Pak temperature control system (Allentown, Allentown, NJ, USA); the temperature of the incoming air was maintained at 28°C. Standard rat chow and tap water were available *ad libitum*. The room was on a 12 h light/dark cycle (lights on at 6:00 A.M.). Each rat was extensively habituated to staying inside a wire-mesh conical confiner. At the time of the experiments, rats weighed 300–450 g.

All rats were implanted with venous catheters. Under ketamine-xylazine-acepromazine (55.6, 5.5, and 1.1 mg/kg, respectively, intraperitoneally) anesthesia and antibiotic (enrofloxacin, 1.2 mg/kg, subcutaneously) protection, a small incision was made on the ventral surface of the neck, left of the trachea. The left jugular vein was exposed, freed from its surrounding connective tissue, and ligated. A silicone catheter (ID, 0.5 mm; OD, 0.9 mm) filled with heparinized (10 U/ml) saline was passed into the superior vena cava through the jugular vein and secured in place with ligatures. The free end of the catheter was knotted, tunneled under the skin to the nape, and exteriorized. The skin wounds on the ventral surface of the neck and on the nape were sutured. The catheter was flushed with heparinized saline on days 1 and 3 after surgery. On day 4, each rat was taken into one of the two experiments.

### Experiment 1: measuring the thermoregulatory response to LPS

To record the colonic temperature (a measure of deep T_b_), each rat was instrumented with a copper-constantan thermocouple (Omega Engineering, Stamford, CT, USA), placed in a confiner, and transferred to a climatic chamber (Forma Scientific, Marietta, OH, USA). The thermocouple was lubricated with Vaseline, inserted into the colon (9–10 cm past the anal sphincter), affixed to the tail with a loop of tape, and plugged into a data logger (model AI-24, Dianachart, Rockaway, NJ, USA). The venous catheter was connected to a polyethelene-50 extension filled with the drug of interest. The extension was then passed through a port in the chamber wall and connected to a syringe. The ambient temperature (T_a_) in the chamber was set to 30.0 or 24.0°C; these conditions are thermoneutral and subneutral (cool), respectively, for rats in the experimental setup used [9]. After a 1-h stabilization period, *E. coli* 0111:B4 LPS (Sigma-Aldrich, St. Louis, MO, USA) suspension (0.5 mg/ml) in pyrogen-free saline (1 ml/kg) or saline was bolus-injected through the extension of the venous catheter. Both the T_b_ and T_a_ were sampled every 2 min throughout the experiment.

### Experiment 2: measuring the c-Fos response

Rats in individual confiners were placed in the climatic chamber, and LPS or saline was injected, as in Experiment 1. At 150 min postinjection, the rats were anesthetized with intravenous ketamine-xylazine-acepromazine (5.6, 0.6, and 0.1 mg/kg, respectively). Through the ascending aorta (right atrium cut), rats were perfused with 50 ml (15 ml/min) of heparinized (10 U/ml) phosphate-buffered saline (PBS) followed by 280 ml (6 ml/min) of cold (4°C) 4% paraformaldehyde (Sigma-Aldrich, catalog number HT50-1-640) in PBS by using a syringe pump (model 53220, Stoelting, Wood Dale, IL, USA). The brains were removed, post-fixed in paraformaldehyde for 6–24 h at 4°C, and transferred to a 20% sucrose solution with 0.02% sodium azide for cryoprotection. The brains were then cut on a freezing microtome into 40 µm sections that were stored at 4°C in PBS with 0.01% sodium azide.

For c-Fos immunochemistry, tissue sections were rinsed with PBS and then incubated: 1) in 0.3% hydrogen peroxide in PBS for 20 min at room temperature; 2) in 0.3% Triton X-100 in PBS for 20 min at room temperature; 3) with rabbit primary polyclonal immunoglobulin G (1∶4,000 in PBS; Santa Cruz Biotechnology, Dallas, TX, USA, catalog number sc-52) for 48 h at 4°C; and 4) with biotinylated goat anti-rabbit immunoglobulin G (1∶400 in PBS; Vector, Burlingame, CA, USA) for 2 h at room temperature. Thereafter, the slides were exposed to avidin-biotin complex (1∶300 in PBS; Vector Elite Kit) for 2 h at room temperature, rinsed, and incubated in 0.02% diaminobenzidine tetrahydrochloride (Sigma-Aldrich), 0.02% nickel (II) sulfate (Wako Chemicals, Richmond, VA, USA), and 0.017% hydrogen peroxide dissolved in 0.1 M Tris-hydrochloride buffer. The tissues were mounted onto gelatin-coated slides and air-dried. Each slide was covered with a glass microcoverslip.

To evaluate the c-Fos expression, we examined the diencephalic and brainstem structures under a microscope (Eclipse E600, Nikon, Tokyo, Japan). Special attention was paid to the structures involved in the neural pathways for thermoregulation and immunoregulation [10], [11]. Only clearly stained cells with a dark reaction product localized in the nucleus were considered c-Fos-immunoreactive neurons. All brains were examined by the same researcher. The initial phase of examination was aimed at identifying a set of brain structures with a robust c-Fos response to LPS (irrespective of the T_a_). The Fos-expression level was compared semi-quantitatively in each brain structure across treatments, and, when necessary, verified by counting the number of Fos-positive neurons per section. Next, we reexamined all LPS-responsive structures in order to identify those that showed a clear difference in the Fos-response to LPS in the cool versus neutral environment. A strong difference was found in two areas only: the paraventricular hypothalamic nucleus (PVH) and the dorsomedial hypothalamic nucleus (DMH); the latter was examined together with the adjacent dorsal hypothalamic area (DA). In the final phase of analysis, we quantified the level of expression by counting Fos-positive neurons in both the PVH and DMH/DA. In each structure, neurons were counted in three consecutive sections from both sides of the slide, and the average of six counts was determined. Photomicrographs were produced by capturing images with a digital camera (HC-2500, Fuji Film, Tokyo, Japan) mounted directly on the microscope.

### Data processing and analysis

Data are reported as mean±SE. The deep T_b_ responses were compared across treatments and time points by a two-way analysis of variance (ANOVA) with repeated measures. The two-way ANOVA was followed by the *post-hoc* Tukey test. The numbers of Fos-immunoreactive cells were compared between experimental groups using the unpaired Student's *t*-test. The differences were considered significant at *P*<0.05.

## Results

### Experiment 1: the thermoregulatory response to LPS

**Figure 1:**
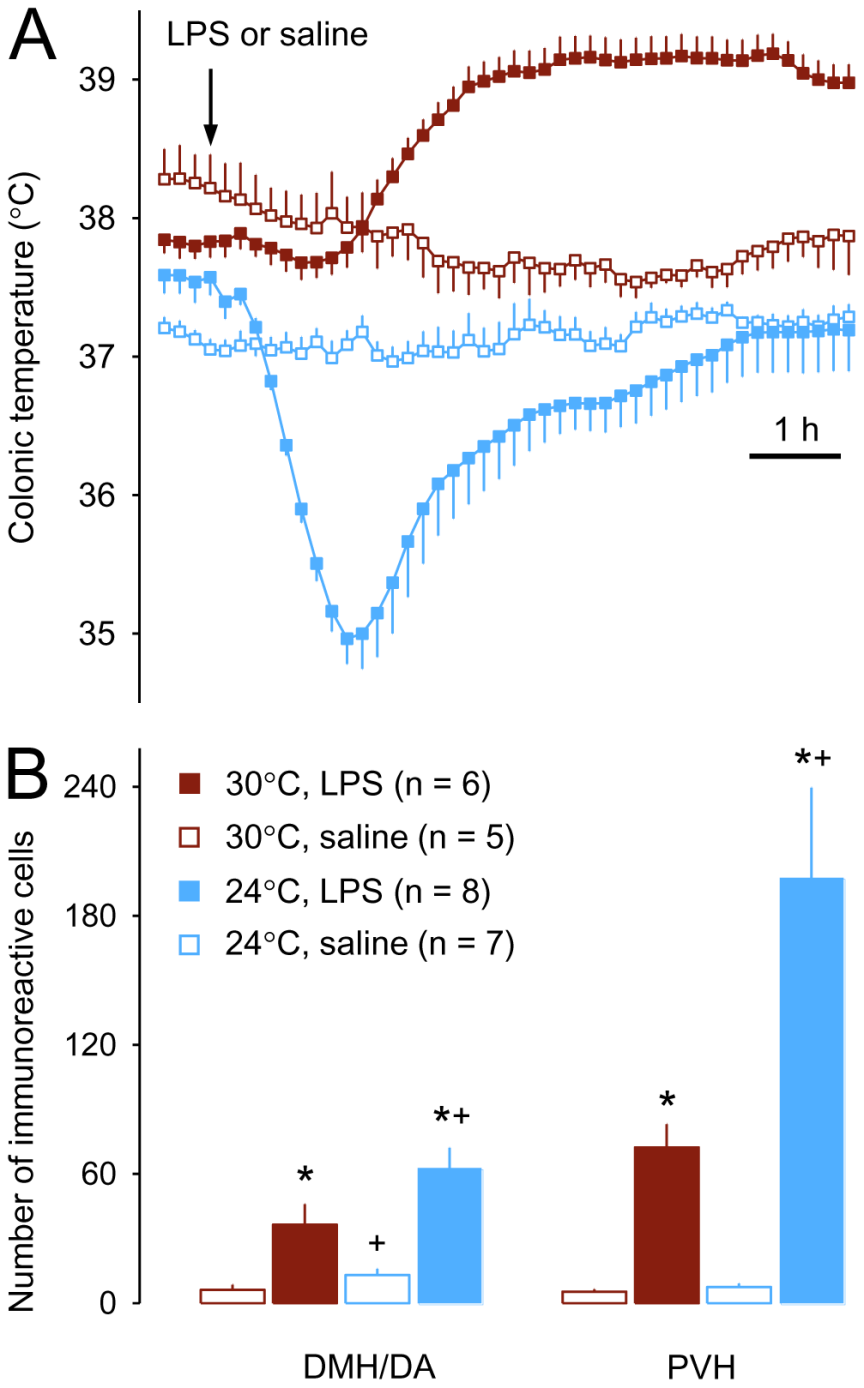
The deep T_b_ response (A) and the hypothalamic (PVH and DMH/DA) c-Fos responses (B) of rats to intravenous LPS (0.5 mg/kg) or saline (1 ml/kg) in a thermoneutral (T_a_ of 30°C) or cool (T_a_ of 24°C) environment. All T_b_ curves differed from each other with high levels of significance (not marked). For c-Fos responses, significant differences (*P*<0.05) are marked as * (compared to saline at the same T_a_) or ^+^ (compared to the same treatment at 30°C).

As expected [12], rats had a ∼0.5–1.0°C higher basal T_b_ at a neutral T_a_ (30°C) than at a subneutral T_a_ (24°C); saline did not alter the T_b_ at either T_a_ (Fig. 1A). In the neutral environment, intravenous LPS (0.5 mg/kg) induced a marked febrile response with the T_b_ reaching a plateau (∼1.3°C above the basal level) at ∼180 min and remaining at that level until the end of the experiment (Fig. 1A). In contrast, the same dose of LPS administered in the cool environment produced a profound hypothermic response with a nadir (∼2.5°C below basal T_b_) at ∼90 min. The observed T_b_ changes were dependent on both the experimental treatment [F (3,989) = 35.767; *P*<0.001] and time [F (45,989) = 15.055; *P*<0.001)]. At thermoneutrality, the T_b_ of febrile, LPS-treated rats differed from that of saline-treated controls during the period 150–420 min post-injection (*P*<0.05). In the cool environment, the T_b_ of hypothermic, LPS-treated rats differed from that of saline-treated controls during the period 50-220 min post-injection (*P*<0.05). Rats treated with LPS at 24°C had a significantly lower T_b_ than rats treated with the same dose of LPS at 30°C during the period 20–420 min (*P*<0.05).

### Experiment 2: LPS-induced Fos expression

**Figure 2:**
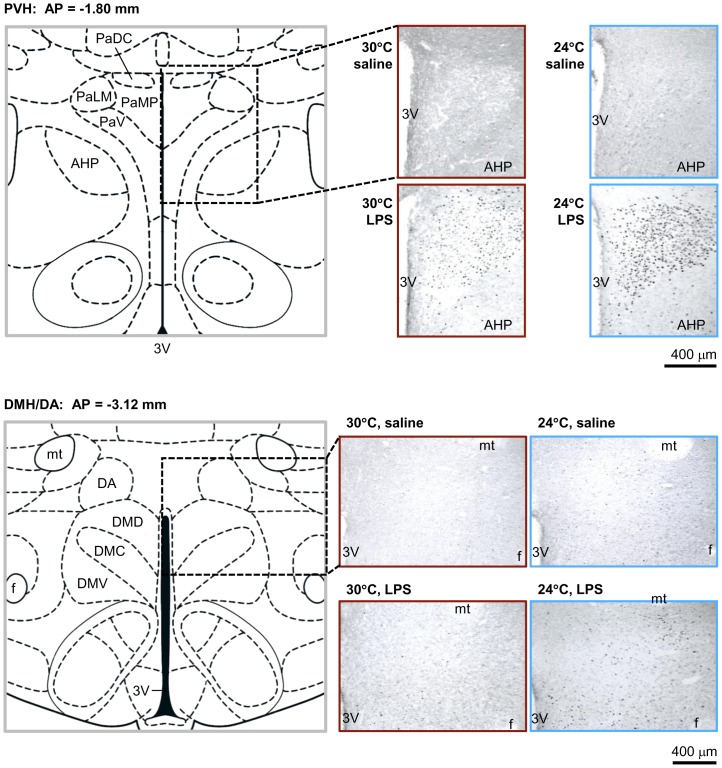
Fos expression in the PVH and DMH/DA areas. Schematics from the Paxinos and Watson [36] atlas and representative photomicrographs of coronal sections from the PVH [the anterior-posterior (AP) coordinate of −1.80 mm from Bregma] and DMH/DA area (AP of −3.12 mm) are shown. The treatment [intravenous LPS (0.5 mg/kg) or saline] and T_a_ (30 or 24°C) are indicated. Photomicrographs of brain sections from rats treated at 30°C are framed in red; sections from rats treated at 24°C are framed in blue. Fos-immunoreactive nuclei can be seen as black dots. For the PVH, the following substructures are marked in the schematic: the dorsal cap (PaDC), the lateral magnocellular and medial parvocellular subnuclei (PaLM and PaMP, respectively), and the ventral aspect (PaV); the third ventricle (3V) and the posterior aspect of the anterior hypothalamic area (AHP) are marked for orientation. For the DMH, the dorsal, compact, and ventral aspects (DMD, DMC, and DMV, respectively) are marked; the DA is also shown. The mammilothalamic tract (mt), fornix (f), and 3V are shown as “landmarks.” The section marked “24°C, LPS” includes only a narrow ventral portion of the mt.

Saline-treated rats presented very limited c-Fos immunoreactivity in all diencephalic and midbrain areas studied, irrespective of the T_a_. The only area that had clearly different Fos expression in saline-treated rats at 24°C, as compared to 30°C, was the DMH/DA. The number of Fos-positive neurons in the DMH/DA area was 6.2±2.4 at 30°C and 13.1±2.7 (111% higher) at 24°C [*t* (10) = 1.891; *P*<0.05; Figs. 1B and 2].

The febrile response to LPS at 30°C was associated with increased Fos expression in most brain areas studied. In the diencephalon, these areas included the *organum vasculosum* of the *lamina terminalis*, the median preoptic nucleus, ventromedial preoptic region, supraoptic nucleus, PVH, anterior hypothalamic area, lateral hypothalamus, and the DMH/DA area, as well as the bed nucleus of the *stria terminalis*. In the PVH, Fos expression was found throughout the entire nucleus, including both magnocellular and parvocellular subnuclei (Fig. 2). Brainstem areas that expressed c-Fos during LPS fever included the posterior pretectal nucleus, ventrolateral periaqueductal gray, lateral parabrachial nucleus, *area postrema*, and nucleus of the solitary tract. Due to the observational nature of our analysis, we were likely to miss brain areas in which the c-Fos response to LPS was less robust.

At 24°C, LPS generally induced a c-Fos expression pattern that was very similar to that seen at 30°C. No brain structure showed a markedly stronger response to LPS in the thermoneutral environment, as compared to the cool environment. Two areas showed a strong increase in the LPS-induced c-Fos expression at 24°C, as compared to 30°C: the PVH and the DMH/DA. Again, due to the nature of this analysis, we might have missed some areas in which the T_a_-dependent difference in the c-Fos response to LPS was less pronounced.

The PVH and DMH/DA areas were subjected to a systematic quantitative analysis. Compared to the 72.4±10.8 Fos-immunoreactive cells found in the PVH of LPS-treated rats at thermoneutrality, the PVH of LPS-treated rats exposed to the cool environment showed a 173% increase [197.8±41.7 cells; *t* (12) = 2.481; *P*<0.05; Figs. 1B and 2]. Similarly, the DMH/DA area of LPS-treated rats exposed to the cool environment had 73% more Fos-positive neurons (62.8±9.4), as compared to LPS-treated rats exposed to the neutral environment [36.4±9.5 cells; *t* (12) = 1.803; *P*<0.05; Figs. 1B and 2].

## Discussion

In agreement with earlier reports [8], [13], [14], we found that the same dose of LPS produced two different thermoregulatory responses in rats exposed to different thermal environments. At a neutral T_a_ of 30°C, rats responded to the high LPS dose used in the present study with fever; at a subneutral T_a_ of 24°C, they developed hypothermia. Both fever and hypothermia are thought to be mediated by the brain; an argument in favor of the brain mediation being that both responses recruit a variety of thermoeffectors, including behavioral [5]. However, the neural substrate of the T_a_-sensitive fever-hypothermia switch is unknown.

Our main finding is that two hypothalamic structures, the PVH and the DMH/DA, showed a substantially higher level of LPS-induced Fos expression at 24°C (when the thermoregulatory response to LPS was hypothermia) than at 30°C (when the response was fever). Similar results, but with different doses of LPS, were reported by Elmquist *et al.* [15]. They found that fever caused by a low dose (5 µg/kg) was associated with induction of the c-Fos protein in both PVH and DMH/DA neurons, whereas marked hypothermia caused by a higher LPS dose (125 µg/kg) was accompanied by a much stronger Fos response in both structures. Based on these findings, it is tempting to propose that PVH and DMH/DA neurons are involved in the circuit for the fever-hypothermia switch.

One hypothetical scenario of how the thermoregulatory response switches from fever to hypothermia was proposed by Ivanov *et al.* [16]. According to this scenario, the T_a_ affects the thermoregulatory response to LPS by changing the distribution of blood flow: directing more flow to the skin and less to the viscera at a higher T_a_ (thermoregulatory skin vasodilation) and directing more flow to the viscera and less to the skin at a lower T_a_ (thermoregulatory skin vasoconstriction). Due to different patterns of blood flow distribution, LPS circulates through different vascular beds and is preferentially delivered to different tissues. By acting on different receptors in different vascular beds and tissues, LPS can be speculated to induce different sets of secondary pro-inflammatory mediators. For example, LPS-induced hypothermia was reported to depend on products of cyclooxygenase (COX)-1 [8], whereas LPS fever is well-known to depend on products of COX-2 [17], [18]. Even though both COX isoforms catalyze the same reaction, they are preferentially coupled with different terminal prostaglandin synthases, thus initiating synthesis of different prostaglandins [17], [19]. Hence, depending on the T_a_, LPS can trigger the production of different mediators, and different mediators can activate different neural structures to drive different thermoregulatory responses. We are currently testing this hypothesis in one of our laboratories.

Another scenario, which has not been studied yet, is that cold and warmth signals from the skin may interact with inflammatory signals. Such an interaction may occur in the periphery, *e.g.*, at thermoreceptors. For instance, the transient receptor potential melastatin-8 channel, which functions as a cutaneous cold sensor for several autonomic and behavioral effectors in the thermoregulation system [20], can be activated by both temperature signals and inflammatory (*e.g.*, bradykinin and histamine) signals [21]. Integration of thermal and inflammatory signals may also occur in the brain, and several hypothalamic structures are well-positioned to constitute such integration sites. For example, prostaglandin E_2_, which can play the role of a systemic mediator of inflammation [22], [23], acts on preoptic neurons (that are involved in normal thermoregulation) to trigger fever [11], [24].

There is also abundant literature showing that both the PVH and DMH/DA serve as integration sites for a variety of stimuli. Both are involved in the control of autonomic thermoeffectors [11], [25]–[29], as well as neuroendocrine and behavioral responses [30]–[32], and the PVH also receives inflammation-related signals [33], [34]. However, the information that is currently available is insufficient to delineate the entire circuit of the fever-hypothermia switch. Nor does it allow for differentiation between various scenarios of how the switch functions.

Interestingly, the PVH and DMH/DA are the same two areas that have been shown by Almeida *et al.* [35] to mediate LPS-induced cold-seeking behavior, which is one of the effectors of LPS-induced hypothermia [6]. However, the Almeida *et al.* [35] findings cannot explain our current results. Almeida *et al.* [35] have found that the cold-seeking response to LPS requires the integrity of neuronal bodies in the DMH and neuronal fibers of passage (but not neuronal bodies) in the PVH, whereas the present and several other studies (*e.g.*, the study by Elmquist *et al.* [15]) show the induction of c-Fos in the bodies of PVH neurons. Moreover, cold-seeking behavior did not contribute to the development of LPS-induced hypothermia in the present study, because rats were not allowed to choose their preferred thermal environment. In this experimental setup, the main effector mechanism of LPS hypothermia is a decrease in the threshold T_b_ for cold-induced thermogenesis – not the initiation of cold seeking [6]. Although the DMH is involved in the control of both LPS-induced cold-seeking behavior [35] and thermogenesis [29], [32], these two functions are likely controlled by different populations of neurons (Wanner S. P., Shimansky Y. P., Almeida M. C., Oliveira D. L., Eales J. R., Coimbra C. C., and Romanovsky A. A., in preparation). Activation of DMH/DA neurons that control thermogenesis increases the T_b_, whereas activation of DMH neurons that control the cold-seeking behavior brings it down. Our present finding of the increased c-Fos expression in the DMH/DA area of rats treated with saline at 24°C, as compared to 30°C, agrees with the role of DMH/DA neurons in the control of thermogenesis.

In conclusion, our results indicate that the recruitment of neurons in the PVH and DMH/DA into the response to bacterial LPS depends on the T_a_. This finding suggests that these hypothalamic areas may be involved in the neural circuit responsible for switching the deep T_b_ response to the inflammatory stimulus from febrile to hypothermic. Neither other parts of this circuit nor the mechanisms by which the circuit functions are currently known.
